# The Role of Major Biomarkers of Stress in Atrial Fibrillation: A Literature Review

**DOI:** 10.19102/icrm.2023.14025

**Published:** 2023-02-15

**Authors:** Saira Rafaqat, Sana Rafaqat, Simon Rafaqat

**Affiliations:** ^1^Department of Zoology, Lahore College for Women University, Lahore, Punjab, Pakistan; ^2^Department of Biotechnology, Lahore College for Women University, Lahore, Punjab, Pakistan; ^3^Department of Business, Forman Christian College (A Chartered University), Lahore, Punjab, Pakistan

**Keywords:** Atrial fibrillation, pathogenesis, physiological stress, psychological stress, stress biomarkers

## Abstract

Numerous studies have reported that physical or emotional stress can provoke atrial fibrillation (AF) or vice versa, which suggests a potential link between exposure to external stressors and AF. This review article sought to describe in detail the relationship between major stress biomarkers and the pathogenesis of AF and presents up-to-date knowledge on the role of physiological and psychological stress in AF patients. For this purpose, this review article contends that plasma cortisol is linked to a greater risk of AF. A previous study has investigated the association between increased copeptin levels and paroxysmal AF (PAF) in rheumatic mitral stenosis and reported that copeptin concentration was not independently associated with AF duration. Reduced levels of chromogranin were measured in patients with AF. Furthermore, the dynamic activity of antioxidant enzymes, including catalase as well as superoxide dismutase, was examined in PAF patients during a period of <48 h. Malondialdehyde activity, serum high-sensitivity C-reactive protein, and high mobility group box 1 protein concentrations were significantly greater in patients with persistent AF or PAF compared to controls. Pooled data from 13 studies confirmed a significant reduction in the risk of AF related to the administration of vasopressin. Other studies have revealed the mechanism of action of heat shock proteins (HSPs) in preventing AF and also discussed the therapeutic potential of HSP-inducing compounds in clinical AF. More research is required to detect other biomarkers of stress, which have not been reported in the pathogenesis of AF. Further studies are required to identify their mechanism of action and drugs to manage these biomarkers of stress in AF patients, which might help to reduce the prevalence of AF globally.

## Introduction

Atrial fibrillation (AF), the most common cardiac arrhythmia, is linked to stroke, heart failure, and death and has caused a significant global health burden that has been predicted to be a growing health issue. Older age and increasing obesity rates, which are strongly related to and may be responsible for AF, may be driving an increase in the incidence of the disease. Obesity, hypertension, diabetes mellitus, sleep apnea, coronary artery disease, heart failure, and alcohol or tobacco use are among the known risk factors for AF that may be reversible.^1^ AF is expected to affect 6–12 million people in the United States by 2050 and 17.9 million people in Europe by 2060. Being a major risk factor for ischemic stroke, AF results in severe morbidity and mortality as well as an enormous economic burden.^2^

Risk factors can vary frequently when accompanied by stress and negative emotions; for instance, those who frequently experience chronic stress report higher rates of smoking, drinking, weight gain, and inactivity. Patients’ symptoms of AF become worse as a result of associated risk factors. Stress has been linked to an increased risk of heart failure, arrhythmogenesis, acute myocardial infarction, and hypertension and has a direct impact on the neuronal, endocrine, autonomic, and immunological systems as well as an indirect one via lifestyle alterations that accelerate the development of cardiovascular diseases (CVDs). When a person believes that internal or external pressures are greater than their ability to adapt, stress is felt. The prevalence of psychological stress in people with AF is increasing, but the pathogenesis remains unknown.^3^

Similarly, the major trigger of cardiac arrhythmias is stress, which exerts profound effects on the electrophysiology of the cardiac rhythm and cardiomyocytes.^4–6^ The impact of the cardiovascular system through the autonomic nervous system is due to physiological and psychological stressors. While stressors vary, properties of the stress response at the level of the cardiovascular system (collectively referred to as autonomic cardiovascular responses) are similar and can be studied independently from the properties of specific stressors.^7,8^ Recently, it has been shown that physical or emotional stress can provoke AF and vice versa.^9–14^ Stress cannot be measured directly, but several substances in the blood can serve as surrogate parameters for stress.^15^

A biomarker is a characteristic that may be scientifically quantified and assessed as a predictor of a pathological or physiological process or as a pharmacological reaction to a therapeutic intervention.^16^ In the same way, Dhama et al. reported on various stress biomarkers, including cortisol, copeptin, malondialdehyde (MDA), chromogranin A (CgA), α-amylase, secretory immunoglobulin A, and more.^17^ Similarly, Noushad et al. detailed potential diagnostic indicators of biomarkers of chronic stress, such as cortisol, adrenocorticotropic hormone, brain-derived neurotrophic factor, catecholamines, glucose, hemoglobin A1c, triglycerides, cholesterol, prolactin, oxytocin, dehydroepiandrosterone sulfate, C-reactive protein (CRP), and interleukin-6 and -8.^18^ Djuric et al. has also described the biomarkers of psychological stress in health disparities research.^19^ This review article focuses only on a few stress biomarkers—specifically, cortisol, copeptin, CgA, superoxide dismutase (SOD) and catalase, MDA, arginine vasopressin (AVP), and heat shock proteins (HSPs)—and their role in AF and highlights the role of physiological as well as psychological stress in the pathogenesis of AF.

## Methods

Several studies have reported the relationship between physiological as well as psychological stress and the progression and development of AF. In the same context, there are numerous biomarkers of stress, but this review article focuses on just a few major stress biomarkers— including cortisol, copeptin, CgA, SOD and catalase, MDA, AVP, and HSPs—in AF and also explains the role of physiological and psychological stress in the pathogenesis of AF.

For this purpose, Google Scholar, PubMed, and ScienceDirect were used to review the literature. April 30, 2022, was the last date of the literature search, and many keywords were used to search the literature, including “stress biomarkers,” “physiological stress,” “psychological stress,” “atrial fibrillation,” and “pathogenesis.” The language of the articles was restricted to English, but we did not set a limit on the publication date. Importantly, this article seeks to summarize the current level of understanding of the topic, as well as reviews of previously published studies, rather than reporting new facts or analysis of the topic.

## The role of major stress biomarkers in the pathogenesis of atrial fibrillation

Although biomarkers have been categorized based on several factors, including traits, applications, genetics, and molecular biology techniques, they can also have a dual nature or perform several functions, making them suitable for a variety of classifications. They might be imaging biomarkers or non-imaging biomarkers depending on their properties.^16^ This review article only focuses on a few major stress biomarkers, including cortisol, copeptin, CgA, SOD and catalase, MDA, AVP, and HSPs, and their role in the pathogenesis of AF, as explained in **Table 1**.

### Cortisol

In the human body, cortisol is a steroid hormone vital for the stress response that regulates a wide range of homeostatic functions. Unregulated cortisol levels can contribute to metabolic pathophysiology.^20^ The risk factor for CVDs is stress^21^; during stress, cortisol is the major hormone released, and its levels are elevated through activation of the hypothalamic–pituitary–adrenal (HPA) axis.^22^ Equally important, Sapolsky et al.^23^ reported that elevated cortisol levels provoke extensive physiological responses like homeostasis maintenance by inducing vasoconstriction as well as sodium retention and energy mobilization, including promoting blood glucose, followed by a breakdown of proteins and fat. Elevated cortisol levels result in a loss of appetite and weight loss; if prolonged, they can promote overeating and weight gain.^24^ A positive link has been found between cardiovascular medication usage as well as type 2 diabetes mellitus and hair cortisol concentrations.^25^

Studies by Rosman et al. and Fransson et al. have also provided evidence supporting a potential link between exposure to external stressors and AF.^26,27^ Additionally, the study by Larsson et al. assessed whether plasma cortisol was causally related to AF using a 2-sample Mendelian randomization design; notably, these authors found that a 1 standard-deviation increase in genetically predicted plasma cortisol was linked to a greater risk of AF (odds ratio [OR], 1.20; 95% confidence interval [CI], 1.06–1.35), but the link was attenuated when adjusting for genetically predicted systolic blood pressure and waist circumference (OR, 0.99; 95% CI, 0.72–1.38).^20^

### Copeptin

The hypothalamic–pituitary axis system releases copeptin, which is a polypeptide and a pre-prohormone, along with neurophysin II as well as vasopressin. Copeptin mainly regulates electrolyte and water balance as it is a C-terminal derivate of AVP and has a diagnostic role in cardiorenal dysfunction. Even though vasopressin is a major hypothalamic stress hormone that circulates throughout the body, its use as a stress biomarker is challenging due to its pulsatile release, rapid clearance, and instability in plasma.^28^ Also, copeptin is a co-component of pre-prohormone released as a more stable protein in an equimolar ratio to vasopressin, which can be finely employed for assessing the individual stress level in comparison to cortisol.^29^

In the same context, Avci et al. investigated the association between increased copeptin levels and paroxysmal AF (PAF) in rheumatic mitral stenosis (MS). In the PAF group, copeptin (6.9 vs. 4.0 pmol/L, *P* < .001) levels were significantly higher than those in the control group. Multivariable logistic regression analysis revealed copeptin level (OR, 2.81; 95% CI, 1.30–5.29; *P* < .001) to be an independent predictor of PAF. In conclusion, the authors reported that a greater risk of developing AF was associated with higher copeptin and high-sensitivity CRP (hs-CRP) levels in patients with mild/moderate rheumatic MS.^30^

Elsewhere, the study by Lang et al. revealed that an elevated level of copeptin was linked to a greater risk of cardiovascular events, including stroke and death, at mid-term follow-up in patients with non-valvular AF, which could make it useful for risk stratification.^31^ In contrast, Arbault-Biton et al. reported that copeptin concentration was not independently associated with AF duration.^32^

### Chromogranin A

CgA is present in the secretory granules of different neuroendocrine tissues and is an acidic protein prohormone recognized as a marker of mental stress.^33^ It is primarily stored in the vesicles of the adrenal glands and released into the circulation along with catecholamines through exocytosis.^34^ The level of CgA is higher in normal subjects at night and lower in the morning.^35^ Meanwhile, the study by Broedbaek and Hilsted^36^ found that CgA is an important biomarker in diabetes. In the same way, recent studies have evaluated CgA as a valuable biomarker in various stressful diseases, including neuroendocrine tumors,^37^ cardiovascular disorders,^38,39^ atopic dermatitis,^40^ ulcerative colitis,^41^ and diabetes mellitus.^42^ Nickel et al. also reported that CgA levels have been shown to increase after running a marathon. This, however, appears to be stress caused by the marathon rather than cardiac burden.^43^

Various studies have shown that the circulating level of CgA is influenced by CVDs, which are also known risk factors for AF and include heart failure, acute coronary syndrome, and hypertension.^44–47^ Nonetheless, no data are available on CgA in the context of AF. Although reduced levels of chromogranin were found in patients with AF, results suggested that CgA levels do not directly support the hypothesis that AF is stress-related or vice versa. Given that the adrenergic system has a huge impact on the development of AF, the authors expected higher CGA levels as a surrogate for elevated sympathoadrenergic tone.^48,49^

Lackermair et al. explored the relationship between arrhythmias and stress, noting that the plasma levels of previously described stress parameters were altered in highly symptomatic patients with AF per se and patients undergoing ablation therapy by pulmonary vein isolation (PVI). In addition, CgA was lower in AF patients compared to healthy controls. Over time, patients without AF after PVI showed an increase in CgA level, while no change was observed in patients with AF after PVI.^49^

### Superoxide dismutase and catalase

In AF, increased oxidative stress could have resulted in the upregulation of pro-oxidant enzymes, including NADPH oxidase, or the downregulation of circulating antioxidant systems such as superoxide dismutase (SOD) or glutathione peroxidase 3 (GPx3), which led to an increased concentration of reactive oxidant species. Markers of oxidative stress seem to have a prognostic role in cardiovascular events in AF.^50^ In healthy workers, SOD and catalase are both antioxidant enzymes that have been studied in the context of stress, and research revealed that SOD levels are elevated in people who work in the evening as well as those doing night shifts.^51,52^

In another study, Negreva et al. examined the dynamic activity of antioxidant enzymes, including catalase and SOD, in PAF patients over a duration of <48 h. The authors observed the activity of SOD, as well as that of catalase, which was greater in PAF patients than controls during hospitalization. Moreover, this difference was maintained 24 h after rhythm regularization and, even 24 days after the restoration of sinus rhythm, the activity of catalase remained increased.^53^

In the same way, the study by Michałek et al. reported that serum CuZn-SOD activity and serum total antioxidant capacity were significantly lower in their study groups compared to in control animals; specifically, they found that catalase activity was significantly higher and plasma GPx activity was significantly lower in dogs with chronic heart failure and AF compared to those with chronic heart failure only, sinus rhythm, or control dogs.^54^

### Malondialdehyde

The MDA activity and serum hs-CRP and high mobility group box 1 protein (HMGB1) concentrations were significantly greater in patients with persistent AF or PAF than in controls. In the patient group, the HMGB1 concentration was significantly positively correlated with MDA activity (*r* = .535) and negatively correlated with SOD activity (*r* = −.491). MDA, SOD, hs-CRP, and HMGB1 were significant independent predictors of AF.^55^

### Arginine vasopressin

AVP is a neuropeptide hormone produced by the hypothalamus and secreted from the posterior lobe of the pituitary gland. AVP is the primary activator of the HPA axis and a vasoconstrictor as well as an antidiuretic that is also being actively studied as a stress biomarker.^56,57^ Additionally, AVP has a behavioral role and is a neural regulator of numerous social behaviors, including affiliation, aggression, and pair bonding.^58^ De Kloet et al. previously identified an elevated concentration of plasma AVP in human patients with post-traumatic stress disorder compared to both traumatized and healthy non-traumatized controls.^59^

In this context, Huang et al. found that congestive heart failure and AVP V1 receptor antagonists coexist with AF and could be used to treat hyponatremia in heart failure. AVP is directly involved in the regulation of the electrophysiological properties of the pulmonary vein, calcium homeostasis, and the identification of the underlying mechanisms.^60^ In the same way, Weiss et al. stated that centrally liberated AVP elevated sympathetic outflow to the cardiovascular system and could increase the risk of arrhythmia and sudden death.^61^ The relationship between AVP and AF has not yet been elucidated. Other studies reported that patients with postoperative vasoplegic shock with an increased AF occurrence rate more frequently experienced new AF with prolonged AVP therapy.^62,63^ McIntyre et al. did find that the addition of vasopressin to catecholamine vasopressors compared to catecholamines alone was linked to a lower risk of AF. Pooled data from 13 studies confirmed a significant reduction in the risk of AF related to the administration of vasopressin.^64^

Vasopressin could contribute to AF reduction by avoiding the adrenergic stimulation caused by catecholaminergic vasopressors. This potential could be confirmed by the fewer number of patients emerging with AF or shorter AF durations in patients and the smaller rate of consequences detected.^65–68^

### Heat shock proteins

HSPs are molecular chaperones that form highly conserved protein families across different species of animals and have multiple physiological roles. They include various critical proteins, such as HSP60, HSP70, and HSP90; among these, HSP70 is the most prominent, with significant effects on diverse biological systems as well as therapeutic potential. The study by Hecker and McGarvey showed that environmental stress acts as a predisposing factor for the synthesis and secretion of various HSPs at greater concentrations. Such stresses include inflammation and infection; during its lifetime, the cell is exposed to various toxic substances, exercise, and a dearth of water. This is the reason why these proteins are also termed stress proteins. Moreover, various HSPs are induced in response to short-term stress, including osmotic stress, heavy metal toxicity, and ecological stress from pollutants.^69^ HSPs play a significant role in the protection of cellular proteostasis, including function, protein expression, and degradation in cells.^70^ Amongst the minor HSPs, HSPB1 seems to have unusual significance in chaperoning the proteostasis of cardiomyocytes by stabilizing the contractile proteins and stopping degradation.^71–74^

Other studies have measured levels of the mitochondrial chaperones mortalin and HSP60 in AF patients. In their work, Schäfler et al. observed a 2.5-fold increase in HSP60 expression in chronic AF patients compared to sinus rhythm patients.^75–77^ Additionally, Baler et al. reported that HSPs were upregulated during diseases, such as early-stage AF, as well as stress, especially through the activation of heat shock transcription factor-1.^78^ Then, the study by Yang et al. indicated that the expression of both HSP27 and heat shock cognate protein 73 may be associated with different stages of AF and that HSP60 also may be associated with the degree of atrial myolysis.^79^ Moreover, both HSP70 and anti-HSP70 antibodies could be linked to the development of AF and AF recurrence after catheter ablation.^80^

Remarkably, HSPB1 levels were found to be increased in atrial tissue samples (from the right atrial appendage [RAA] and left atrial appendage [LAA]) of patients with PAF; however, these levels are exhausted in patients with longstanding persistent AF,^71^ indicating that low RAA and LAA HSPB1 levels are associated with AF progression. Furthermore, lower HSPA1 tissue levels correlated with postoperative AF (PoAF) in a study of patients who underwent coronary artery bypass grafting.^81^

Other studies have shown the mechanism of action of HSPs in preventing AF and also discussed the therapeutic potential of HSP-inducing compounds in clinical AF and the potential of HSPs as biomarkers to discriminate between the recurrence of AF and various stages of AF after treatment.^82^ In the same way, Sakabe et al. suggested that HSP induction could be a useful new anti-AF intervention in patients with coronary artery disease.^83^

Another study observed that elevated HSP27 expression in PAF patients could serve to protect myocytes from myolysis and limit the development of persistent AF. Geranylgeranylacetone (GGA) is a drug that is pharmacologically important for the induction of HSPs, which could represent a novel therapeutic approach to AF.^71^ Hu et al. also reported that HSPB1 levels in serum from patients treated with ablative therapy predicted AF recurrence.^84^ Another study reported that AF is closely connected to atherosclerotic burden, and fluctuations in levels of several HSPs have been related to an increased occurrence of AF in stressed cardiomyocytes after ischemia–reperfusion injury. The modulation of HSP expression may be a valuable therapeutic strategy in the management of AF and atherosclerosis.^85^

Recently, Brundel et al. examined whether HSPs prevent AF and attenuate the promotion of AF in both cellular and animal experimental models. In the human studies, it was suggested that HSPs could play a protective role against progression from PAF to chronic, persistent AF. Novel therapeutic approaches for the prevention of atrial remodeling and manipulation of the HSP system could be helpful, which would further contribute to the maintenance or restoration of tissue integrity as well as contractile function and also delay the progression toward chronic AF and/or improve the outcome of cardioversion.^86^

HSPs are a kind of serum biomarker that could enable AF staging and reveal patients at risk for AF recurrence and PoAF. Therefore, van Marion et al. concluded that HSPA5 RAA and HSPD1 RAA and LAA levels change in persistent stages of AF. Both RAA HSPA1 and HSPA5 levels are linked to the progression of PoAF. Furthermore, HSPB1 RAA and HSPA5 LAA levels could predict AF recurrence in patients who underwent arrhythmia surgery. However, HSP levels in serum cannot discriminate AF stages from controls, or predict PoAF or AF recurrence after treatment.^87^

Van Marion et al. also identified novel GGA derivatives with improved physicochemical properties compared to GGA. GGA derivatives, particularly GGA*-59, enhance HSP expression, resulting in the prevention of and restoration from tachypacing-induced remodeling, substantiating their role as novel therapeutics in clinical AF.^88^

## Discussion

**Figure 1:**
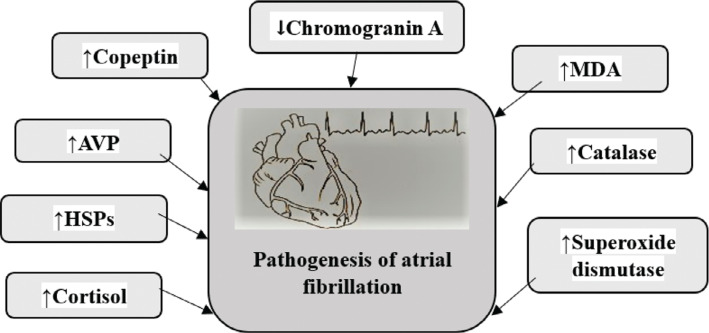
The overall presentation of each biomarker of stress in the pathogenesis of atrial fibrillation. *Abbreviations:* AVP, arginine vasopressin; HSP, heat shock protein; MDA, malondialdehyde.

This review article discusses the role of major biomarkers of stress in the pathogenesis of AF as explained in **Figure 1**. Stress is a recognized risk factor capable of causing acute cardiovascular events, a contributor to several life-threatening medical diseases, and a major contributor to many social issues. Globally, the burden of stress is rising, and, with it, so too is the interest in creating efficient stress-monitoring systems to support proactive and connected health initiatives, particularly given the expanding use of wearable sensor technology. The development of linked wearable devices to monitor stress and take timely action to halt the advancement of stress-related medical disorders has been made possible by the recent development of small and flexible biosensors. Anxiety, depression, pathological stress, and other diseases linked to stress have all notably increased in recent years. Stress generally harms the physical, mental, and emotional health of a person. Chronic stress specifically increases the risk of obesity, diabetes, stroke, and CVD.^89^

In the same context, stress is a common psychophysiological reaction that the body produces in response to unfavorable, demanding, and unpleasant situations or stressors. Literature supports the negative effects of stress on the human brain, which have been known for >50 years. It has been documented that the neuroanatomical alterations linked to stress, such as brain atrophy brought on by prolonged stress, cause differential responses and have an effect on cognition and memory.^18^

In populations who deal with the ongoing stress of daily living, psychological stress can contribute to health inequities. Psychological stress has been proven to have an impact on several biomarkers. Allostatic load (the cumulative burden of chronic stress and life events), a summary indicator of the accumulated biological burden of repeated attempts to adjust to daily stress, is one of these biomarkers. The hypothalamic–pituitary axis, the sympathetic nervous system, and the cardiovascular system are all obstructed by the allostatic load, which has an impact on the immune system through 2-way signaling pathways. Additionally, there is increasing evidence that psychological stress can increase oxidative stress levels and DNA damage, possibly through elevated inflammatory states. Researchers are only beginning to understand how race, genotype, gene expression, and the capacity to effectively attenuate stress response interact.^19^

Similarly, the sympathetic nervous system and the HPA axis are both physiological processes that are triggered by psychological stress. When encountering conditions that cause fear or anxiety, these systems act in coordination. When triggered, the hypothalamus releases corticotropin-releasing hormone, which travels through the hypophyseal portal circulation to the anterior pituitary gland and then through projections from the paraventricular nucleus of the hypothalamus to the locus ceruleus.^90^

The elevated concentration of perceived stress has been linked to prevalent AF in the Reasons for Geographic and Racial Differences in Stroke (REGARDS) study.^91^ In contrast, the study by Svensson et al. was the first of its kind to investigate the interaction between a genetic risk score strongly predictive of AF and stress. However, these authors’ findings did not directly support a relationship between the progression of incident hospital-diagnosed AF and chronic psychological stress and did not find any interaction between the genetic risk of AF and stress.^92^

Elsewhere, Lane et al. examined 70 patients with AF in which the predominant effective response after a diagnosis of AF was anxiety.^93^ A case–control study of 101 AF patients and 97 hypertensive patients in sinus rhythm reported that AF patients have higher levels of depression and anxiety, negatively impacting their quality of life.^94^

Meanwhile, the study by Ong et al. revealed that reduced quality of life in AF patients may be related to depression in women but not in men.^95^ Depressive symptoms also have been related to an increased recurrence of AF after electrical cardioversion to normal sinus rhythm.^96^ However, only data from the Framingham Offspring Study have revealed that increased levels of tension, anxiety, anger, and aggression precede the development of AF in men but not in women.^97^

In 2 patients with normal echocardiograms and coronary angiograms, PAF was triggered by psychological stress. Both patients were free of metabolic abnormalities and neither were alcoholics nor had consumed ethanol before the start of AF. AF may be caused by various processes, which remain to be explored.^98^

In general, positive emotions may be beneficial, while negative emotions have been connected to the onset of AF. A few large-scale studies have observed the relationship between psychosocial stressors that can cause these emotions and the development of AF. Financial stress, traumatic life experiences, and neighborhood stress were all significantly greater among women with AF. After accounting for cardiovascular risk factors, socioeconomic level, and psychosocial state, only traumatic life events remained substantially linked to AF (OR, 1.37; 95% CI, 1.19–1.59). Therefore, the authors’ extensive cross-sectional data suggest a possible connection between traumatic life events and AF in older women.^99^

Numerous studies have highlighted the elevated rate of anxiety among AF patients as a result of their poor quality of life; nevertheless, nothing is known regarding the possibility that anxiety could cause AF. The study by Severino et al. highlighted any potential pathophysiological connections between anxiety disorders and AF. It was suggested that anxiety can act as a trigger for AF, produce an arrhythmogenic substrate, and alter the autonomic nervous system. Understanding how mental disorders affect AF risk may result in the making of new treatments.^100^

The prevalence of psychological stress with AF is increasing, but the pathogenesis is still unknown. Stress and AF appear to interact complexly in both directions, with a bidirectional link between both conditions. The immunological and autonomic nerve systems, which are important initiators and potentiators of AF, are modulated by stress. Anxiety, psychological distress, and suicidal thoughts all increase as a result of AF. The fourth pillar of managing AF has recently been identified as lifestyle adjustment, with stress reduction existing as a potentially reversible risk factor and potential intervention target. Additional research into anxiolytic and antidepressant therapy, mindfulness-based stress reduction, and yoga is required, as these are potential treatment alternatives to decrease stress as part of AF care.^3^

Also, in the postoperative period, anxiety symptoms were linked to AF. Hospital staff should take note of worry as it relates to AF following heart surgery in acute cardiac care and cardiac rehabilitation settings. However, it is unclear how worried thoughts affect a patient, how AF symptoms were sensed, or whether anxiety feelings frequently come before AF.^101^

Inflammation and AF are strongly related as patients with AF have significantly higher levels of inflammatory biomarkers. Unexpectedly, the overall prevalence of neither AF nor any of its subtypes was associated with higher levels of urinary F(2)-isoprostanes, a sensitive indicator of systemic oxidative stress in vivo.^102^

Finding protein biomarkers linked to the incidence of AF might help with pathophysiology research, risk assessment, and the development of new AF treatments. The authors looked at the relationships between 85 protein biomarkers and new cases of AF and linked a higher incidence of incident AF to lower levels of insulin-like growth factor 1 and higher levels of insulin-like growth factor binding protein 1 and N-terminal B-type natriuretic peptide.^103^ Elsewhere, Wu et al. suggested that new findings would add to the growing evidence suggesting that enhanced oxidative stress has a significant pathogenic role in PoAF.^104^

The considerable correlation between natriuretic peptides and AF was verified, and mid-regional proadrenomedullin and sensitive troponin I ultra as novel biomarkers representing vascular function, inflammation, and myocardial injury were related to AF. Prospective studies must look into whether employing these biomarkers might improve the risk prediction of AF beyond clinical risk factors.^105^

This review article has highlighted the function of physiological biomarkers in chronic stress and psychological stress biomarkers and outlined their predictive and therapeutic values. These psychological stress biomarkers can be very helpful in understanding the pathogenesis of AF. More research is required to detect other biomarkers of stress that have not been reported in the pathogenesis of AF. Also, further studies will be required to identify their mechanism of action and drugs to manage these biomarkers of stress in AF patients, which might be supportive to reduce the prevalence of AF globally.

## Conclusions

This review article concludes that major stress biomarkers (cortisol, copeptin, CgA, SOD and catalase, MDA, AVP, and HSPs) play a significant role in the pathogenesis of AF. Also, up-to-date knowledge about the role of physiological and psychological stress in the pathogenesis of AF has been presented. The identification of more biomarkers of stress is required for the development of relevant therapeutic approaches in AF patients.

## Figures and Tables

**Table 1:** Summary of the Studies Explaining the Role of Different Major Biomarkers of Stress in the Pathogenesis of Atrial Fibrillation

Study	Number of Patients	Stress Biomarker(s)	Main Finding(s)
Larsson et al., 2021^20^	12,597	Plasma cortisol	Two-sample Mendelian randomization design study suggests a positive link between plasma cortisol and AF risk, likely mediated through systolic blood pressure and waist circumference
Avci et al., 2021^30^	29	Copeptin	Investigated the association between increased copeptin levels and PAF in rheumatic MS
Lang et al., 2013^31^	253	Copeptin	Elevated levels of copeptin were linked to a higher risk of cardiovascular events, including stroke and death, at mid-term follow-up in patients with non-valvular AF and could be useful for risk-stratification
Arbault-Biton et al., 2021^32^	98	Copeptin	Copeptin concentration was not independently associated with AF duration
Lackermair et al., 2017^49^	96	Chromogranin A	Chromogranin A concentrations were lower in AF patients compared to healthy controls (13.8 vs. 25.6 ng/mL, *P* < .01)
Negreva et al., 2014^53^	51	Catalase, superoxide dismutase	PAF was characterized by significantly increased activity of SOD and catalase even in the early hours of clinical manifestation of the disorder, which then slowly decreased with the restoration of sinus rhythm
Wu et al., 2013^55^	86	hs-CRP, HMGB1, MDA	Serum hs-CRP and HMGB1 concentrations and MDA activity were significantly higher in patients with persistent AF (n = 33) or PAF (n = 53) versus controls (n = 30)
Cheng et al., 2018^62^	1,156	AVP	Patients with postoperative vasoplegic shock with increased AF occurrence more frequently experienced new AF with prolonged AVP therapy
Schäfler et al., 2000^76,77^	14	HSP60	The study revealed a 2.5-fold increase in HSP60 expression in chronic AF patients compared to sinus rhythm patients
Yang et al., 2007^79^	24	HSP27, HSC73, HSP60	The expressions of HSP27 and HSC73 could be linked to different stages of AF, and that of HSP60 also could be linked to the degree of atrial myolysis
Kornej et al., 2013^80^	67	HSP70	HSP70 and anti-HSP70 antibodies could be linked to the development of AF as well as AF recurrence after catheter ablation
van Wijk et al., 2021^82^	—	HSP	Mechanism of action of HSPs in preventing AF
Sakabe et al., 2008^83^	35 mongrel dogs	HSP	HSP induction could be a useful new anti-AF intervention for patients with coronary artery disease.
Lin et al., 2017^85^	—	HSP	Modulation of HSP expression may be a valuable therapeutic strategy in the management of AF and atherosclerosis

*Abbreviations:* AF, atrial fibrillation; AVP, arginine vasopressin; HMGB1, high mobility group box 1 protein; hs-CRP, high-sensitivity C-reactive protein; HSC, heat shock cognate protein; HSP, heat shock protein; MDA, malondialdehyde; MS, mitral stenosis; PAF, paroxysmal atrial fibrillation; SOD, superoxide dismutase.
